# Nitroreductase Activites in *Giardia lamblia*: ORF 17150 Encodes a Quinone Reductase with Nitroreductase Activity

**DOI:** 10.3390/pathogens10020129

**Published:** 2021-01-27

**Authors:** Joachim Müller, Manfred Heller, Anne-Christine Uldry, Sophie Braga, Norbert Müller

**Affiliations:** 1 Department of Infectious Diseases and Pathobiology, Vetsuisse Faculty, Institute of Parasitology, University of Bern, Länggass-Strasse 122, CH-3012 Bern, Switzerland; 2 Proteomics & Mass Spectrometry Core Facility, Department for BioMedical Research (DBMR), University of Bern, Freiburgstrasse 15, CH-3010 Bern, Switzerland; manfred.heller@dbmr.unibe.ch (M.H.); anne-christine.uldry@dbmr.unibe.ch (A.-C.U.); sophie.lagache@dbmr.unibe.ch (S.B.)

**Keywords:** anaerobic metabolism, drug susceptibility, functional assays, mass spectrometry, protein chromatography

## Abstract

The intestinal diplomonadid *Giardia lamblia* is a causative agent of persistent diarrhea. Current treatments are based on nitro drugs, especially metronidazole. Nitro compounds are activated by reduction, yielding toxic intermediates. The enzymatic systems responsible for this activation are not completely understood. By fractionating cell free crude extracts by size exclusion chromatography followed by mass spectrometry, enzymes with nitroreductase (NR) activities are identified. The protein encoded by ORF 17150 found in two pools with NR activities is overexpressed and characterized. In pools of fractions with main NR activities, previously-known NRs are identified, as well as a previously uncharacterized protein encoded by ORF 17150. Recombinant protein 17150 is a flavoprotein with NADPH-dependent quinone reductase and NR activities. Besides a set of previously identified NRs, we have identified a novel enzyme with NR activity.

## 1. Introduction

The intestinal diplomonadid *Giardia lamblia* is a causative agent of persistent diarrhea in humans and various animal species [1,2,3,4]. Currently, chemotherapy of giardiasis is mostly based on nitro drugs, in particular metronidazole [5,6,7], with nitazoxanide as a potential alternative [8]. The prevailing model implies that electrons provided by pyruvate ferredoxin oxidoreductase (PFOR) reduce nitro compounds to toxic intermediates, thereby causing nitrosative stress [9,10,11]. Moreover, we have characterized two nitroreductases (NRs) with N-terminal ferredoxin and C-terminal flavine-NAD(P)H reductase domains, namely the nitazoxanide-binding protein NR1 [12,13] and a homologous protein referred to as NR2 [14]. In addition, the *Giardia* genome contains another homologous gene encoding the reductase domain only (NR family protein, referred to as NR3 [15]). In functional assays, NR1, NR2, and NR3 are good quinone reductases, but are modest NRs with only residual activities on nitro drugs [15,16]. A useful system to further characterize these proteins is the overexpression in *Escherichia coli* BL21, a strain that is susceptible to metronidazole and various other antibiotics. NR1 overexpressors become more susceptible, NR2 overexpressors become more resistant to metronidazole, and NR3 overexpressors become more resistant to tetracycline [15,16]. Other examples for a reductase with residual activities on nitro compounds are thioredoxin-reductase [10,17], glycerol-3-phosphate dehydrogenase [18], the nitric oxide (NO) reducer flavohemoglobin [19,20], and the strong O_2_ scavenger, but weak NO reducer A-type flavo- or flavodiiron protein [21,22], both involved in the antioxidant stress response [23,24,25]. Depending on the background genotype and the nitro drugs involved, resistance formation to nitro drugs in clones of various strains may be correlated with a downregulation of PFOR or NR1, or with an upregulation of radical scavengers [26,27]. Moreover, NAD(P)H oxidase, the predominant detoxifying system of O_2_ in *G. lamblia*, has residual NR activities [28].

In the present manuscript, we ask the following questions: (1) Are these previously characterized enzymes the only ones responsible for nitroreduction? (2) Can we identify other proteins with this function? (3) Are these proteins isolated or associated? To answer these questions, we fractionated cell-free extracts of trophozoites by size exclusion chromatography. To assay NR activity, we chose NAD(P)H as electron donors, and 7-nitrocoumarin as a substrate. In fact, 7-nitrocoumarin is a suitable substrate for assaying the nitroreductase activity of the previously characterized NR1 and NR2 [16], as well as for assaying NR activities of crude extracts [29]. Peaks with NR activities are pooled and analyzed by mass spectrometry. The previously uncharacterized protein encoded by ORF17150 identified by this approach is characterized.

## 2. Results

### 2.1. Size Distribution of NAD(P)H-Dependent NR Activities in G. lamblia Extracts

**Figure 1:**
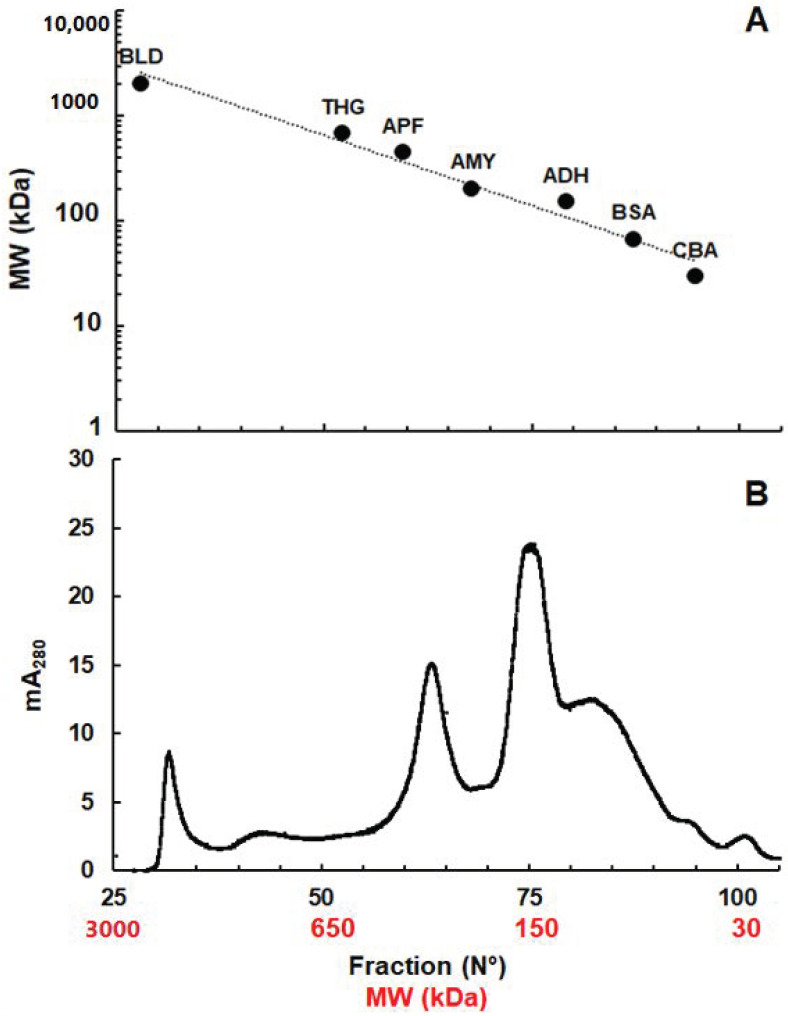
Separation of soluble proteins from *G. lamblia* crude extracts by size exclusion chromatography. (**A**) Calibration curve using a marker panel, namely blue dextran (BLD, 2000 kDa), thyreoglobulin (THG, 670 kDa), apoferritin (APF, 443 kDa), amylase (AMY, 200 kDa), alcohol dehydrogenase (ADH, 150 kDa), bovine serum albumin (BSA, 66 kDa), and carboanhydrase (CBA, 29 kDa); (**B**) *G. lamblia* proteins. Size exclusion chromatography was performed as described in the Materials and Methods section.

Separation of *G. lamblia* crude extracts by size exclusion chromatography gave three major protein peaks at around 2000 kDa, 300 kDa, and 150 kDa (Figure 1).

**Figure 2:**
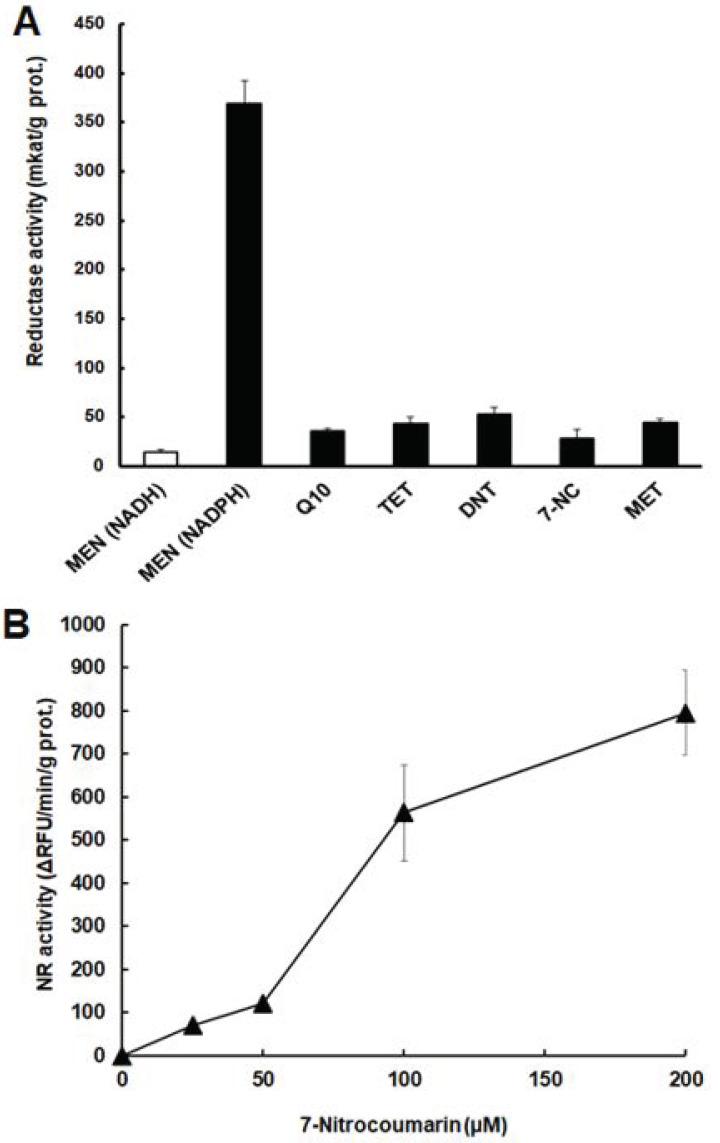
Nitroreductase activities in soluble protein fractions from *G. lamblia* crude extracts separated by size exclusion chromatography. (**A**) Activity measured via MTT reduction; **(B)** activity measured via direct 7-nitrocoumarin reduction. Size exclusion chromatography and functional assays were performed as described in the Materials and Methods section. The assays were performed with NADH (black symbols) or NADPH (white symbols) as electron donors. Blanks without 7-nitrocoumarin were subtracted.

In the next step, NR activities were measured in the fractions obtained as described above using either NADH or NADPH as electron donors and MTT as a final electron acceptor. Moreover, NR activity was directly measured using 7-nitrocoumarin as a substrate (Figure 2).

Major NR activity eluted in fractions 60–67, thus, not at their relative native molecular weights, but between 330 to 230 kDa. Using MTT as the final acceptor, NADPH-dependent activity was predominant by far (Figure 2A). Only by assaying NR activity via the formation of the fluorescent 7-aminocoumarin could NADH-dependent activities higher than NADPH-dependent activities be detected in fractions 71 (180 kDa) and 94 (43 kDa), as shown in Figure 2B.

### 2.2. Identification of Proteins in Fractions with Reductase Activities

In order to investigate whether proteins with confirmed NR activities matched the protein fractions with the corresponding activities, the fractions with NR activities as identified above (see Figure 2A,B), namely 32–34, 61–63, 64–66, 70–72, and 93–95, were pooled, and the proteins were identified by shotgun MS (raw dataset in Table S1) and are listed in Table 1.

Moreover, fractions 44–46 with only background NR activities were analyzed. The complete list of identified proteins is given as supplemental data (Table S2).

Enzymes with NR activities confirmed by previous studies were identified in all pools, except pools 1 and 6.

**Figure 3:**
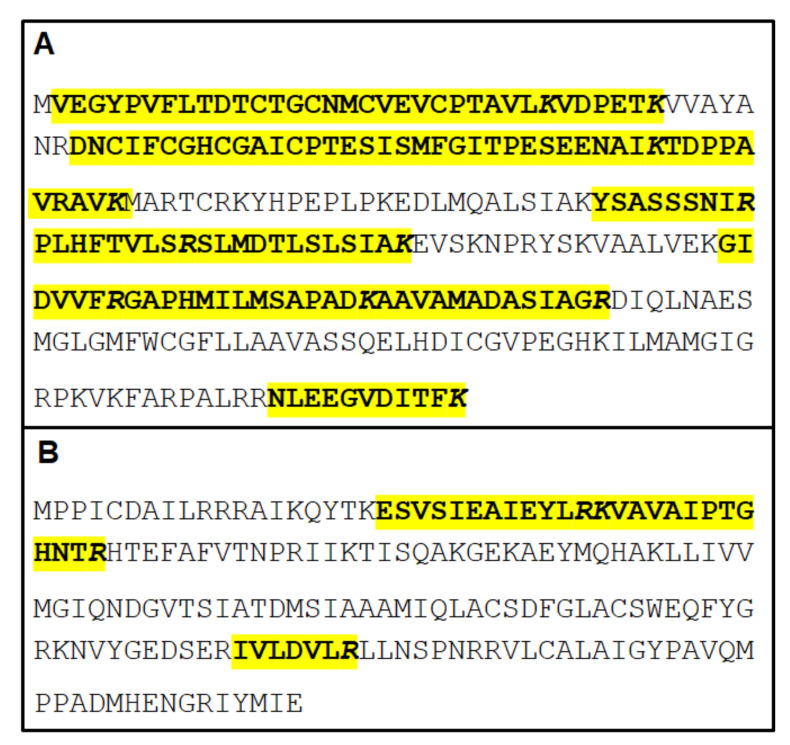
Identified peptides of the nitroreductase Fd-NR2 (“NR1”, **A**) and the nitroreductase family protein (“NR3”, **B**) within the corresponding primary sequences.

The weak NO reducer A-type flavoprotein was identified in pools 1 to 5, thus also in pool 2, without detectable NR activities. Glycerol-3-phosphate dehydrogenase, NAD(P)H oxidases, and thioredoxin reductase, proteins with confirmed NR activities, were identified together with the two pyruvate flavodoxin oxidoreductases in pools 3 to 5. In addition, pool 5 contained the well-characterized NR1 [12] and the NR family protein NR3 [15], a quinone reductase with residual NR activity. The sequences with the identified peptides of both proteins are presented in Figure 3.

This is interesting, because in previously published proteome datasets [27,30], NR3 could not be detected in *G. lamblia* WBC6.

Pool 6 did not contain proteins with confirmed NR activities.

### 2.3. Identification of a Putative NADPH Oxidase Encoded by ORF 17150

**Figure 4:**
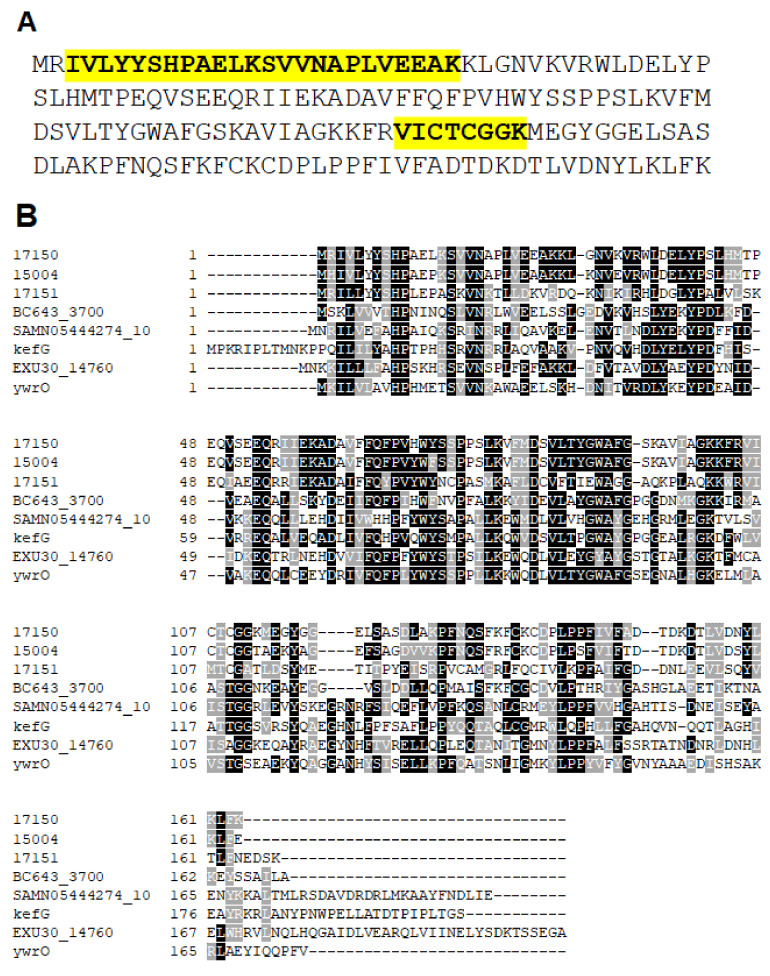
Identification of a protein encoded by ORF 17150 in pools 5 and 6. (**A**) Identification of peptides within the amino acid sequence predicted by the ORF. (**B**) Alignment of ORF 17150 to homologous ORFs in the genomes of *Giardia* WBC6 (15004, 17151) and in the genomes of various bacteria (for a more detailed description, see text).

Interestingly, pools 5 and 6 contained a previously uncharacterized quinone reductase homologous, namely, a protein encoded by ORF 17150, as identified by the peptides shown in Figure 4A. ORF 17150 encodes an 18 kDa polypeptide with two homologs in the *Giardia* genome, namely 15004 (86.6% identity) and 17151 (44.2% identity). Two overlapping peptides (SVVNAPLVEAAK+SVVNAPLVEAAKK) were detected for 15004 (Table S1). Peptides encoded by ORF 17151 could not be detected in our dataset. Furthermore, 17150 aligned to the putative NADPH quinone reductase (BC643_3700) from *Mangrovibacterium diazotrophicum* (43% identity), the Kef-type potassium/proton antiporter accessory protein (SAMN05444274_104409) from *Mariniphaga anaerophila* (34.9% identity), the glutathione-regulated potassium-efflux system ancillary protein (kefG CFU_0384) from *Collimonas fungivorans* (39.1% identity), the flavodoxin family protein (EXU30_14760) from *Shewanella maritima* (36.1% identity), and the general stress protein 14 (ywrO BSU35990) from *Bacillus subtitlis* (40.1% identity). Alignments are depicted in Figure 4B.

### 2.4. The Polypeptide Encoded by ORF 17150 Is a NADPH-Dependent Quinone Reductase with Nitroreductase Activity

**Figure 5:**
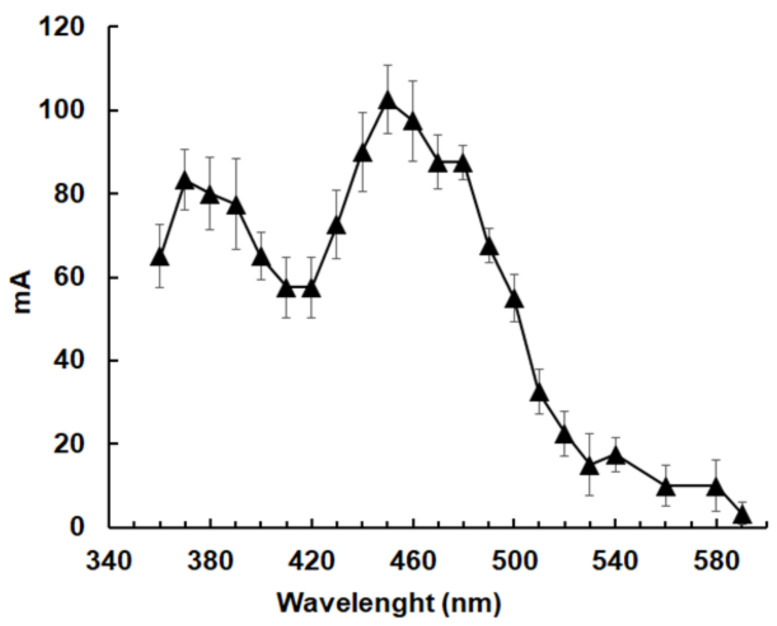
Absorption spectrum of His-Tag purified protein P17150 obtained after heterologous expression in *E. coli* BL21(DE3).

In order to characterize the polypeptide encoded by ORF 17150 in more detail, we cloned the corresponding sequence into *E. coli* and purified the resulting His-Tag fusion protein by affinity chromatography. The purified protein had a characteristic spectrum with an optimum at 450 nm, indicative for flavoproteins (Figure 5).

**Figure 6:**
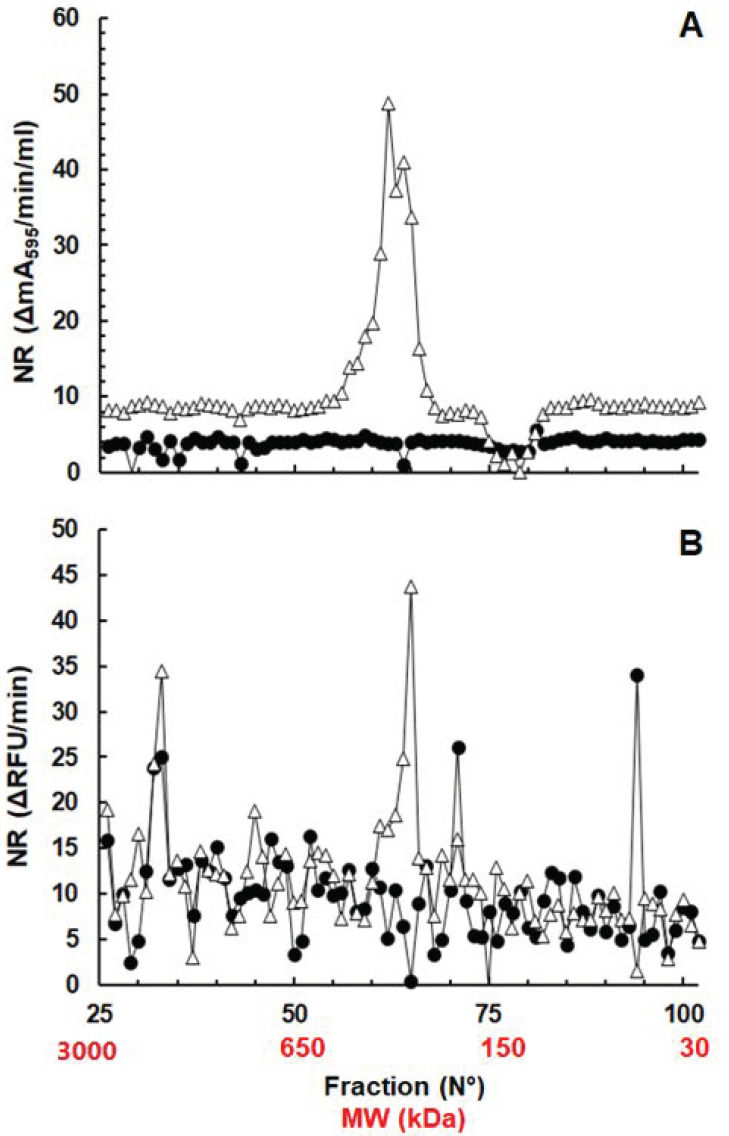
Functional assays with recombinant protein P17150. (**A**) Assays based on the oxidation of NADH (white bar) or NADPH (black bars) with menadione (MEN), coenzyme Q10 (Q10), tetracycline (TET), dinitrotoluene (DNT), 7-nitrocoumarin (7-NC), or metronidazole (MET) as electron acceptors; **(B)** 7-nitrocoumarin reduction assay (with NADPH as the electron donor) based on the formation of 7-aminocoumarin as an end product.

In functional assays using menadione as an electron acceptor and NADH or NADPH as electron donors, the recombinant P17150 largely preferred NADPH to NADH. Other quinones, such as coenzyme Q and tetracycline, could be used as electron acceptors, but with much lower specific activities. Moreover, the nitro compounds dinitrotoluene, 7-nitrocoumarin, and metronidazole could be used as electron acceptors (Figure 6A). These findings prompted us to perform an NR assay with P17150 based on the reduction of 7-nitrocoumarin to 7-aminocoumarin as a fluorescent end product. The recombinant protein 17150 clearly reduced 7-nitrocoumarin to 7-aminocoumarin in a concentration-dependent manner (Figure 6B).

**Figure 7:**
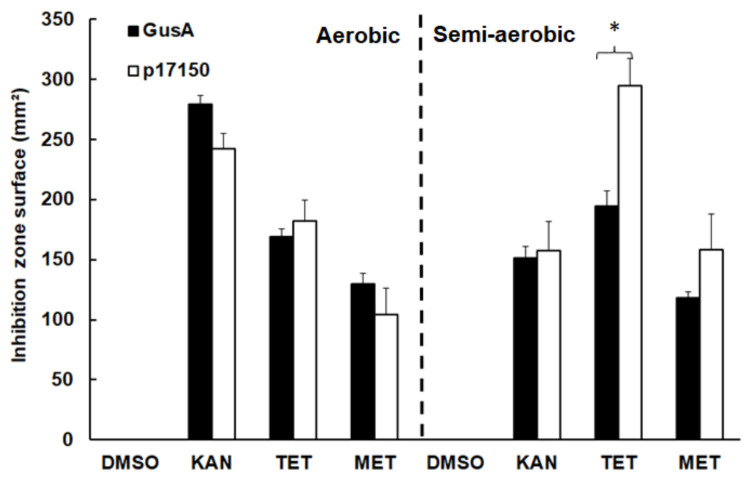
Susceptibility of *E. coli* BL21(DE3) expressing glucuronidase A as a control (GusA), or protein 17150 (p17150) to tetracycline (TET), metronidazole (MET), or kanamycin (KAN). Lines were plated, discs containing the drugs were added, and plates were incubated under aerobic or semi-aerobic conditions. After 16 h, diameters of inhibition zones were determined, the surfaces of inhibition zones were calculated, and the values were processed as described in the Materials and Methods section. Mean values ± standard errors correspond to four independent replicates. Values marked by an asterisk are significantly different between p17150 overexpressors and GusA controls (two-sided *t*-test; *p* < 0.01).

In order to investigate whether the overexpression of protein 17150 affects the susceptibility to tetracycline (a quinone) and/or to metronidazole in *E. coli*, BL21 was compared to a glucuronidase A (GusA) overexpressing control strain. Semi-aerobically grown *E. coli* expressing protein 17150 had significantly higher susceptibility to tetracycline than the GusA strain control. In the presence of metronidazole, no significant effects were observed. Susceptibility to the non-quinone antibiotic kanamycin was not significantly affected in any of the bacterial cultures tested. Under aerobic conditions, the susceptibilities to TET were not affected (Figure 7).

## 3. Discussion

In *G. lamblia* crude extracts, major NR activity was found between 330 and 170 kDa. Four proteins with previously identified NR activities, namely two NAD(P)H oxidases, glycerol-3-phosphate dehydrogenase, and thioredoxin reductase, are present in the fractions throughout this range. Since the molecular weights of the corresponding polypeptide sequences were much smaller (between 35 and 53 kDa), it is very likely that they are associated to each other as homo- or heteromultimers. Both isoforms of PFOR present in the same fractions are potential association partners as well. The nitroreductase NR1 (annotated as Fd-NR2 in the GiardiaDB) with a polypeptide molecular weight of 29 kDa could only be identified in fractions between 190 and 170 kDa. Therefore, an association with other partners is very likely. All proteins interacting with HA-tagged NR1 in a previously published co-immunoprecipitation study [16] were present in pool 5, together with NR1. These proteins are listed in Table 2.

Of particular interest in this context are two glycolysis enzymes, namely fructose-bisphosphate aldolase and phosphoglycerate kinase. Both enzymes are present in all pools with NRs and PFOR, suggesting a channeling of pyruvate, the end product of glycolysis, to the PFOR isoforms, which provide the electrons for the reduction of quinones (most likely the physiological function), as well as for the reduction nitro compounds yielding toxic intermediates. Moreover, the NR1 co-precipitation partner malate dehydrogenase is present not only in pool 5 together with NR1, but also in pools 3 and 4, the major pools with NR activity. Malate dehydrogenase provides electrons from carbon sources different from glucose, such as amino acids, the predominant C sources for *G. lamblia* in axenical culture [31]. The NR1 homologous NR2 was not detected neither in the present study nor in previously published whole cell shotgun MS studies [27,30], nor by immunoblotting [27]. A recently published study on genetic polymorphisms in genes involved in resistance against metronidazole listed malate dehydrogenase as a potential resistance marker besides the “usual suspects”, such as PFORs, ferredoxins, and NRs [32] including the NR2 (GL50803_6175) orthologue DHA2_153380. The corresponding mRNA is clearly detectable [14,29]. The absence of protein signals in MS shotgun and immunoblot analyses could be due to very low translation levels and/or to a rapid degradation of the polypeptide. The role of arginyl-tRNA-synthetase within this association is unclear. Results obtained with the *Plasmodium falciparum* enzyme suggest that it is inactivated by free heme [33]. Similarly, reductases associated with the *Giardia* enzyme could prevent its inactivation by bile pigments or other xenometabolites. The normalized leading protein data of the proteins of interest mentioned in this paper are included in Table S3.

Another ORF identified in this study is 17150. The protein encoded by this ORF was detectable in pools 5 and 6. By functional assays, we identified this protein as a quinone reductase largely preferring NADPH to NADH. It is a flavoprotein without N-terminal ferredoxin domain. With an estimated concentration of 12.5 µM for protein 17150 in fraction 2, the FAD or FMN molecular ratio amounts to 0.7 ± 0.1 flavin molecules per molecule of this flavoprotein. The metabolic function of this novel quinone reductase in *G. lamblia* remains to be elucidated. The closest homologues are found in bacteria, indicative for acquisition by lateral transfer [34], and may be involved in the stress responses, as suggested by expression profiles and in silico phylogenetic studies [35]. Like 17150, the homolog YwrO enzyme is an NADPH-dependent quinone reductase with NR activity [36]. Recombinant *E. coli* overexpressing protein 17150 are more susceptible to tetracycline, but not to metronidazole, under semi-aerobic conditions. Perhaps, under these conditions, tetracycline is reduced to a derivative with a higher toxicity [37]. This differs from previous results obtained with strains overexpressing other quinone reductases, namely NR1 and NR2, which have no effect on tetracycline, but increasing (NR1) or decreasing (NR2) the susceptibility to metronidazole [15,16], and from strains overexpressing NR3, which are less susceptible to tetracycline and more susceptible to metronidazole [15].

Transcription levels of ORF17150 do not exhibit large variations in axenic tachyzoites, but decrease during encystation, as shown in the datasets contained in the GiardiaDB (www.giardiadb.org). In trophozoites collected from mouse intestines, however, 17150 is significantly overexpressed compared to axenic trophozoites [38]. In trophozoites subjected to shotgun mass spectrometry, protein levels of 17150 are in the same range as NR1 levels and, in our study, not affected in nitro drug resistant versus susceptible strains [27]. In another study, 17150 protein levels were, however, decreased in a metronidazole-resistant strain [26].

Since protein 17150 reduces several nitro compounds, in particular 7-nitrocoumarin to 7-aminocoumarin in functional assays, it can be considered as an unspecific NR, for instance NR1, NR3, thioredoxin reductase, and other proteins. Similarly, the well-characterized *E. coli* NRs NfsA [39] and NfsB [40] are multifunctional flavin reductases, and can be used as reporter genes for the quantification of hypoxia in tumor cells [41]. It is, however, unlikely that this novel NR contributes to the NADH-dependent NR activity detected in pool 6. The best candidate for NADH-dependent complete reduction, the nitroreductase NR2, is not detectable, as discussed above. Other proteins may have residual NR activities. Our dataset presented in the Supplementary Materials (Tables S1–S3) will support further research in this field.

Taken together, our results confirm previous studies showing that the reduction of nitro compounds in *G. lamblia* is not performed by a single, predominant NR, but by a set of known and unknown enzymes with (residual) NR activities. This is in agreement with data suggesting that nitro drug resistance is multifactorial [10] and due to metabolic changes, e.g., via pool sizes of cofactors involved in reduction [10,29], rather than to mutations or differential expression of marker genes.

## 4. Materials and Methods

### 4.1. Biochemicals

If not otherwise stated, all biochemical reagents were from Sigma (St. Louis, MO, USA). Dinitrotoluene, menadione, and metronidazole, 7-nitrocoumarin were kept as 100 mM stock solutions in DMSO at −20 °C. Conversely, tetracycline (solubilized in DMSO) and kanamycin (solubilized in H_2_Oad inject.) were kept as 2 mM stock solutions.

### 4.2. Axenic Culture, Harvest, and Storage of G. lamblia Trophozoites

Trophozoites from *G. lamblia* WBC6 were grown under anaerobic conditions in 10 mL culture tubes (Nunc, Roskilde, Denmark) on a modified TYI-S-33 medium, as previously described [42]. Subcultures were performed by inoculating 100 μL of cells from a confluent culture detached by cooling to a new tube containing 10 mL of the culture medium as described [43]. Pellets were washed three times with ice-cold PBS, then counted and stored at −80 °C for subsequent size exclusion chromatography.

### 4.3. Size Exclusion Chromatography

To perform size exclusion chromatography, 3 × 10^7^ tachyzoites were extracted in 1 mL of Tris-Cl^-^ (0.05 M, pH = 7.1) containing 0.15 M of NaCl, 0.05% Triton X 100, and 1 mM of PMSF, followed by centrifugation (10 min, 12,000× *g*, 4 °C). The supernatant containing 3.6 mg/mL of protein was loaded on a Hi-Prep16/60 Sephacryl S-400HR column pre-equilibrated with Tris-Cl^-^ (0.05 M, pH = 7.1) containing 0.15 M of NaCl. The column was pre-calibrated with a gel filtration marker set consisting of blue dextran (2000 kDa), thyreoglobulin (670 kDa), apoferritin (443 kDa), amylase (200 kDa), alcohol dehydrogenase (150 kDa), bovine serum albumin (66 kDa), and carboanhydrase (29 kDa) in 1 mL of equilibration buffer. The flow rate was adjusted to 0.5 mL/min. The chromatography was performed using a BioLogic LP system (Bio-Rad Laboratories, Hercules, CA, USA) according to the instructions of the manufacturer. One ml fractions were collected. Aliquots of 50 µL were stored on ice until further analysis by functional assays, and the remaining material was frozen and stored at −80 °C for subsequent analysis by mass spectrometry.

### 4.4. Proteomic Analysis of the Fractions by Mass Spectrometry

Fractions with NR activity were pooled into five fractions. In addition to one pool containing fractions without NR activity, the pools were analyzed by mass spectrometry. Size exclusion chromatography fractions with NR activity were pooled into five fractions. The pools were concentrated to approximately 200 μL by consecutive centrifugation of 500 μL aliquots over a 10 kDa molecular weight cutoff membrane centrifugal device (Nanosep^®^ with Omega™, PALL Life Scineces, Basel, Switzerland) at 14,000× *g* and at room temperature. For the following steps, the buffer was added onto the filter with subsequent concentration by centrifugation, as described before. First, the buffer was exchanged once with 200 μL of 8M urea and 0.1 M of Tris-Cl^-^ (pH = 8), then, 100 μL of urea buffer containing 0.05 M of dithiothreitol were added, followed by incubation for 30 min at 37 °C and constant mixing with 600 rpm. This step was repeated with 100 μL of urea buffer containing 0.05 M of iodoacetamide. The buffer was then exchanged by three consecutive add-ons of 100 μL of urea buffer, followed by a concentration to about 30 μL. To this concentrate, 120 μL of 0.02 M of Tris-Cl^-^ (pH = 8.0) and 2 mM of calcium dichloride were added, and proteins were digested with the addition of 100 ng of sequencing-grade trypsin (Promega, Dübendorf, Switzerland), and then incubated over night at room temperature. After digestion, the solution above the filter was transferred into a clean 1.5 mL polypropylene reaction vial and acidified with 7.5 μL of 20% (*v/v*) trifluoroacetic acid. After an incubation for 15 min at room temperature, the digest was spun for 1 min at 16,000× *g*, and the cleared supernatant was transferred to a HPLC vial for subsequent nano-liquid reversed phase chromatography coupled to tandem mass spectrometry, as described earlier [30].

### 4.5. Cloning, Expression, and Affinity Purification of the Protein Encoded by ORF 17150

The open reading frame 17150 was amplified from the *Giardia* genome using the primers 17150_F 5′-CACCATGCGTATCGTCCTCTACTAC-3′ and 17150_R 5′-TTACTTAAAGAGC-TTCAAATAGTTATC-3′, resulting in a 499 base pair product. For expression in BL21(DE3), the product was cloned into the *E. coli* His-Tag expression vector system pET151 (Invitrogen pET151 directional TOPO; Thermo Fisher Scientific, Waltham, MA, USA), according to the instructions of the manufacturer. His-Tag purification of the recombinant protein was performed as previously described for NRs [12,14,15].

### 4.6. Functional Assays

NR activity in the fractions obtained by gel filtration was determined with 7-nitrocoumarin (0.1 mM) as a substrate by two different end point assays, namely via reduction of thiazolyl blue tetrazolium (MTT) and by determination of the end product, namely 7-aminocoumarin. Both assays were performed using NADH or NADPH (0.5 mM) as described [16]. Moreover, some assays with the recombinant protein 17150 were performed by continuously measuring the direct reduction of NADH or NADPH (0.15 mM) at 340 nm [12], using the substrates (0.1 mM) as indicated. Blanks without substrates (i.e., direct reduction of MTT or O_2_) were subtracted for each assay.

The spectrum of recombinant protein 17150 was obtained using a NanoDrop™ One Microvolume UV-Vis Spectrophotometer (Thermo Fisher Scientific, Waltham, MA, USA), according to the instructions of the manufacturer.

### 4.7. Drug Susceptibility Tests in E. coli

The drug susceptibility of recombinant *E. coli* BL21(DE3) lines expressing either protein 17150 or GusA were tested by a disc diffusion agar procedure as described [16]. Briefly, bacteria were grown to the stationary phase (OD600nm = 1) in Luria-Bertoni medium (LB) containing 100 µg/mL of ampicillin, and 0.3 mL of suspension was streaked on LB agar plates containing 100 µg/mL of ampicillin. Whatman filter discs (5 mm diameter) were soaked with 7 µL of tetracycline, kanamycin (2 mM), or metronidazole (100 mM) stock solutions. The discs were air-dried for 5 min and placed on the plates. The plates were incubated under aerobic or semi-aerobic (5% O_2_, 10% CO_2_, 85% N_2_) conditions at 37 °C for 24 h. Then, growth inhibition zone diameters were measured, and the inhibition zone around the disc was calculated. For each compound, the values were expressed as a percentage of the mean value of the Gus control. Mean values ± SEs are given for four replicates. Values marked by asterisks are significantly different to the GusA-expressing control (two-sided *t*-test, *p* < 0.01 after correction for multiple experiments).

### 4.8. Statistics

The mass spectrometry data were searched by four database search engines, namely Comet [44], Xtandem [45], MSGF [46], and Myrimatch [47], and processed by the suite of Transproteomics Pipeline [48] tools version 5.1. The following search parameters were used: peptide mass tolerance 10 ppm, fragment mass tolerance 20 ppm, cleavage rule strict trypsin allowing for maximum of three missed cleavages. Allowed modifications were fixed carbamidomethylation of cysteines, variable oxidation of methionines, and acetylation of protein N-termini. The searches were performed on the database concatenated with the reversed sequences as decoys. The Peptide Prophet [49] tool was applied separately on the different search results, then iProphet [50] was used to obtain a probability distribution from all search engines. The results were filtered at a false discovery rate of 0.01, and only identifications that were confirmed by at least two search engines were accepted. Protein inference was performed with the TPP tool Protein Prophet [51], and the results were filtered at a false discovery rate of 0.01. A Normalized Spectral Abundance Factor [52] was calculated for each protein group, using the approach of Zhang and coauthors [53] for the distribution of shared peptides; *t*-tests were performed using the software package R [54].

## Figures and Tables

**Table 1:** Overview of MS analysis of *G. lamblia* proteins separated by gel filtration. Five pools of gel filtration fractions with nitroreductase (NR) activity (see Figure 2) and one pool without detectable activities were analyzed by shotgun MS. Proteins with confirmed oxido-reductase activities are listed with their GiardiaDB ORF numbers and their calculated monomeric molecular weights in kDa. G3PDH, glycerol-3-phosphate dehydrogenase; FP, flavoprotein; LT, lateral transfer; NO, NAD(P)H oxidases; NR1, nitroreductase Fd-NR2; NR3, nitroreductase family protein; PFOR, pyruvate flavodoxin oxidoreductase; TrxR, thioredoxin reductase.

Pool	Fractions	MW Approx. (kDa)	Identified Proteins (Number)	NR Activity	Confirmed Nitroreductases (GiardiaDB ORF - MW)	
1	32–34	2000–1600	15	Yes	No confirmed nitroreductases	
2	44–46	950–840	104	No	A-type FP 10358	45
3	61–63	330–280	405	Yes	A-type FP 10358	45
					G3PDH 16125	119
					NO 9719	47
					NOLT 33769	53
					PFOR 114609	138
					PFOR 17063	132
					TrxR 9827	35
4	64–66	277–245	426	Yes	A-type FP 10358	45
					G3PDH 16125	119
					NO 9719	47
					NOLT 33769	53
					PFOR 114609	138
					PFOR 17063	132
					TrxR 9827	35
5	70–72	190–170	985	Yes	A-type FP 10358	45
					G3PDH 16125	119
					NO 9719	47
					NOLT 33769	53
					PFOR 114609	138
					PFOR 17063	132
					TrxR 9827	35
					NR1 22677	29
					NR3 15307	19
6	93–95	46–41	22	Yes	No confirmed nitroreductases	

**Table 2:** The presence of proteins co-precipitated with the nitroreductase NR1 (“Fd-NR2”, ORF 22677) in a previous study in pools, as detailed in Table S2; nd, not detected. NR1 was found in pool 5 only.

Giardia DB ORF.	Protein Annotation	Pools
10521	Arginyl-tRNA-synthetase	**5**
11043	Fructose-bisphosphate aldolase	3,4,**5**
137716	Axoneme-associated protein GASP-180	2,**5**
17411	TCP1-chaperon-subunit gamma	3,4,**5**
3331	Malate dehydrogenase	3,4,**5**
6175	Nitroreductase family protein fused to ferredoxin domain Fd-NR1 (“NR2”)	nd
7532	Vacuolar ATP-synthase catalytic subunit A	3,4,**5**
90872	Phosphoglycerate kinase	3,4,**5**
9183	Hypothetical protein	2,3,4,**5**

## Data Availability

Not applicable.

## Supplementary Materials

The following are available online at https://www.mdpi.com/2076-0817/10/2/129/s1, Table S1: Mass spectrometry dataset, Table S2: List of proteins identified in pools 1 to 6 obtained by size exclusion chromatography. Table S3. Normalized leading protein data of the proteins of interest mentioned in this study.
